# Evolution of Function in the “Two Dinucleotide Binding Domains” Flavoproteins

**DOI:** 10.1371/journal.pcbi.0030121

**Published:** 2007-07-20

**Authors:** Sunil Ojha, Elaine C Meng, Patricia C Babbitt

**Affiliations:** 1 Department of Biopharmaceutical Sciences, University of California San Francisco, San Francisco, California, United States of America; 2 Department of Pharmaceutical Chemistry, University of California San Francisco, San Francisco, California, United States of America; 3 Institute for Quantitative Biomedical Research, University of California San Francisco, San Francisco, California, United States of America; European Bioinformatics Institute, United Kingdom

## Abstract

Structural and biochemical constraints force some segments of proteins to evolve more slowly than others, often allowing identification of conserved structural or sequence motifs that can be associated with substrate binding properties, chemical mechanisms, and molecular functions. We have assessed the functional and structural constraints imposed by cofactors on the evolution of new functions in a superfamily of flavoproteins characterized by two-dinucleotide binding domains, the “two dinucleotide binding domains” flavoproteins (tDBDF) superfamily. Although these enzymes catalyze many different types of oxidation/reduction reactions, each is initiated by a stereospecific hydride transfer reaction between two cofactors, a pyridine nucleotide and flavin adenine dinucleotide (FAD). Sequence and structural analysis of more than 1,600 members of the superfamily reveals new members and identifies details of the evolutionary connections among them. Our analysis shows that in all of the highly divergent families within the superfamily, these cofactors adopt a conserved configuration optimal for stereospecific hydride transfer that is stabilized by specific interactions with amino acids from several motifs distributed among both dinucleotide binding domains. The conservation of cofactor configuration in the active site restricts the pyridine nucleotide to interact with FAD from the re*-*side, limiting the flow of electrons from the re*-*side to the si-side. This directionality of electron flow constrains interactions with the different partner proteins of different families to occur on the same face of the cofactor binding domains. As a result, superimposing the structures of tDBDFs aligns not only these interacting proteins, but also their constituent electron acceptors, including heme and iron-sulfur clusters. Thus, not only are specific aspects of the cofactor-directed chemical mechanism conserved across the superfamily, the constraints they impose are manifested in the mode of protein–protein interactions. Overlaid on this foundation of conserved interactions, nature has conscripted different protein partners to serve as electron acceptors, thereby generating diversification of function across the superfamily.

## Author Summary

The sequencing of genomes from different species has provided a unique opportunity for comparative analysis and opened the door to a higher level of understanding of living organisms. However, identifying the biochemical functions of the protein products coded by these genes has proved to be a major challenge. Computational methods that have been used to assign functions to such sequences often result in high levels of misannotations. Nature's strategy of evolving new function provides clues for formulating an accurate predictive scheme for functional annotation. Constraints associated with substrate binding properties and chemistry have been shown to be major determinants of guiding the evolution of new function. In this study, the authors have explored the functional and structural constraints imposed by complex cofactors on the evolution of new functions. Analysis of the large “two dinucleotide binding domains” flavoproteins (tDBDF) superfamily using structural comparisons and other bioinformatics approaches shows how structural requirements associated with cofactor reactivity constrain the mode of protein–protein interactions while providing the major route for evolution of functional diversification. The evolutionary framework established in this work may be generally useful for the analysis of functional divergence in other enzyme superfamilies that use complex cofactors.

## Introduction

The large disparity between the number of unique protein folds and the number of unique proteins that exist in biological organisms [1] indicates that nature has utilized a relatively small number of folds to generate a large number of different functions. Nature's strategy for recruiting a protein scaffold to supply a range of different functions provides clues for understanding functional mechanisms at the molecular level and predictive power in assigning molecular functions to genes from the genome projects. Evolving a new function often occurs through divergence from a parental gene; constraints associated with a parent scaffold can be linked to a wide array of properties—including folding, cofactor binding, chemical mechanism, or interactions with substrates—that constrain regions of the gene to evolve at slower rates, giving rise to conserved structural features recognizable from sequence or structural comparisons. Identifying structural features conserved between distantly related proteins can thus allow inference of chemical mechanism, substrate binding properties, or function. For example, several studies have demonstrated that aspects of chemical mechanism, in particular, can constrain evolution of new functions in enzyme superfamilies [2–5]. Members of such mechanistically diverse superfamilies have evolved to catalyze a wide range of overall reactions using a common partial reaction or chemical attribute (see [6,7] and references therein for examples). These partial reactions are mediated by highly conserved structural features in the active site. Identifying the residues that comprise these structural features has been exploited to infer function for new proteins discovered in the genome projects, correct misannotations in sequence databases, and engineer new enzymatic functions [7–9].

Models for the evolution of new functions in enzymes that use complex cofactors (excluding metal ions) may be similar to those previously described for mechanistically diverse enzyme superfamilies in that a common fundamental step in the chemical mechanism, in this case the catalytic role of the cofactor(s), is conserved while substrates, products, and overall reactions may differ substantially. Yet enzyme superfamilies that use complex cofactors are also likely to differ in important ways from cofactor-independent enzymes. In cofactor-independent enzymes, the potential range of evolutionary variation in enzymatic function can be large because amino acids involved in determining specificity or that play a direct role in catalysis are subject to natural drift. In cofactor-dependent enzymes, however, only the apoprotein is subject to natural drift. Moreover, while interactions between cofactor and apoprotein can serve as a source of structural variation, giving rise to the evolution of new functions, the range of potential variation is defined by the catalytic repertoire of a given cofactor.

To assess the functional and structural constraints imposed by complex cofactors on the evolution of new protein functions and to determine the extent to which the chemistry-constrained model of enzyme evolution [6] can be used to predict functional properties in such superfamilies, we have examined the sequence, structure, and functional links among the members of the two dinucleotide binding domains flavoproteins (tDBDF) superfamily. These proteins are involved in many different biological activities, including energy metabolism, apoptosis, maintenance of redox homoeostasis, and cellular signaling [10–16]. Collectively, characterized members catalyze oxidation/reduction of a wide variety of substrates, either small molecules or proteins. All tDBDFs, as the name implies, have in common two dinucleotide binding Rossmann fold domains fused in a single peptide chain. Both domains are required for function, and both are always present in all the members of the superfamily. Each of these domains binds one of the two dinucleotide cofactors, FAD and a pyridine nucleotide, respectively. (A notable exception is the flavocytochrome c sulfide dehydrogenase family, in which the pyridine nucleotide is replaced by hydrogen sulfide.) In most tDBDF superfamily members, the N-terminal domain binds the FAD and the C-terminal domain binds the pyridine nucleotide. Associated with their use of these cofactors, subsets of the tDBDF superfamily have previously been shown to exhibit several conserved sequence motifs associated with cofactor interaction or catalysis. These motifs are distributed across both dinucleotide binding domains [17,18] and include two canonical dinucleotide binding motifs (DBMs) with sequence signatures of GxGxxG/A and three additional motifs, ATG, GxxP, and GD, ordered as DBM_FAD_-ATG-DBM_PYRIDINE NUCLEOTIDE_-GxxP-GD. Previously, for many of these proteins, Vallon demonstrated a strong association between dinucleotide binding domains and these characteristic motifs, and suggested a common origin for all tDBDFs [17]. Detailed reaction mechanisms have been elucidated for several tDBDF enzymes (see Argyrou and Blanchard [19] for a recent review).

**Figure 1:**
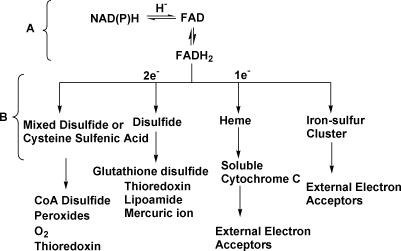
Schematic of Reactions Catalyzed by tDBDF The reductive and oxidative half reactions are shown in brackets with (A) denoting the reductive half-reaction and (B) denoting the oxidative half-reaction. In the oxidative half-reaction, superfamily members can transfer electrons one or two at a time via intermediate acceptors to a variety of different small molecule acceptors or external protein partners.

Although a number of individual members of the tDBDF superfamily have been characterized extensively, large-scale studies have been limited to subsets of these proteins. In a study of sequence–structure relationships among FAD-dependent enzymes, Dym and Eisenberg defined a subset of the tDBDFs that they named Group 1 of the glutathione reductase (GR1) structural superfamily [18]. The GR1 group was subsequently further subdivided by Argyrou and Blanchard into four families, namely flavoprotein disulfide reductases (FDR) 1 through 4, according to the chemical reactions they catalyze [19]. Reactions catalyzed by these (and by extension, all of the additional members of the tDBDF superfamily described here) can be divided into two parts. The first is a reductive half-reaction in which a hydride ion is transferred between a pyridine nucleotide (or hydrogen sulfide in the case of sulfide dehydrogenase) and FAD (Figure 1A). This is followed by an oxidative half-reaction in which two electrons are transferred between FADH_2_ and penultimate acceptors, either one or two electrons at a time (Figure 1B). Enzymes that catalyze transfer of two electrons at a time transfer them to small molecules and proteins via one or multiple cysteine residues. Enzymes that transfer one electron at a time pass them directly to small acceptor molecules, heme or iron-sulfur clusters, presented by interacting proteins. These electrons are further transferred on to the next set of acceptors as shown at the bottom of Figure 1B.

In this work, we have extended those previous studies to identify additional families in the superfamily and to provide a unified view of common structure–function relationships across all of the highly divergent members. Our analysis of the many subgroups and families in the tDBDF superfamily shows multiple connections among sequences that had previously been linked only through pairwise comparisons of a small number of divergent structures. We have linked structural similarities conserved across the entire superfamily with common aspects of their functions and identified structural differences that distinguish functional variations among the subgroups and the families within them. Finally, we show how structural requirements associated with cofactor reactivity have both constrained the location of protein–protein interactions involved in electron transfer and provided an avenue for diversification of function across the many different members in the tDBDF superfamily. The evolutionary framework we have established may be generally useful for the analysis of functional divergence in other superfamilies of enzymes that use complex cofactors.

## Results/Discussion

### Identification and Clustering of tDBDFs

**Figure 2:**
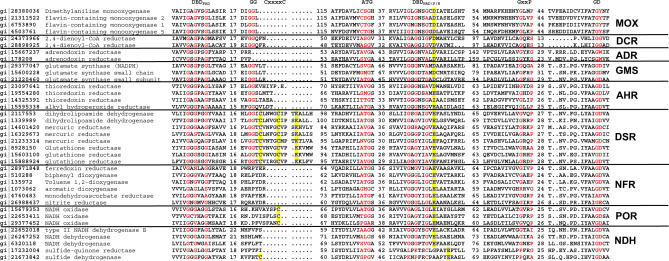
Sequence Alignment of Representative Sequences of Subgroups Containing at Least One Experimentally Verified Enzyme in the tDBDF Superfamily The conserved motifs associated with the superfamily are labeled at the top. The numbers of amino acids separating each motif are shown. Functionally important residues including the cysteine residues of the CxxxxC motif of DSR subgroup, the cysteine sulfenic acid of the POR subgroup, and a cysteine residue that binds FAD covalently in cytochrome c sulfide dehydrogenase are colored in blue and highlighted in yellow. An asterisk * designates the lysine and glutamate residues that stabilize the isoalloxazine and nicotinamide ring complex. Not all motifs are conserved in all subgroups, as discussed in the text.

From initial seed sequences representing most of the tDBDF families, hidden Markov model (HMM)–based searches identified more than 1,650 sequences that belong to the tDBDF superfamily. Only a small minority of these proteins has been experimentally characterized. The functions of ∼9% of these sequences are listed in the National Center for Biotechnology Information database as unknown, and another ∼13% are either annotated with multiple or with unclear descriptions of function, or are clearly misannotated (S. Ojha and P. C. Babbitt, unpublished data). An alignment generated from these sequences shows that all of the tDBDF-specific motifs are conserved and occur in the following order: FAD binding DBM in the N-terminus followed by an ATG motif, and pyridine nucleotide binding DBM, GxxP, and GD motifs. Figure 2 provides an alignment showing these conserved motifs for selected members that have been previously characterized. This alignment is consistent with structure-based alignments, especially with respect to the conserved sequence motifs, and agrees generally with data previously published by Vallon et al. for a small subset of tDBDFs [17].

**Figure 3:**
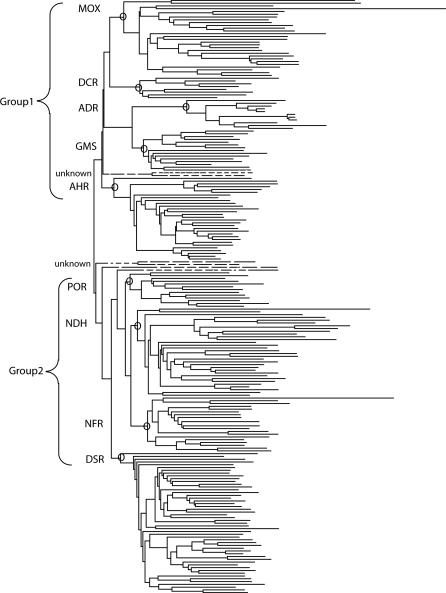
Dendrogram Showing Primary Groups and Subgroups in the tDBDF Superfamily Nodes defining subgroups that contain at least one characterized member are identified by a circle and named using the abbreviations provided in Table 1. Functionally uncharacterized sequences identified in our searches that do not fall into subgroups and that are not listed in Table 1 are shown using dashed lines. For ease of viewing, only a representative set of sequences is shown.

A dendrogram generated from this sequence alignment shows that the tDBDF superfamily can be divided into two major groups, each consisting of several clusters (Figure 3). Two small clusters and a few singletons totaling 22 sequences did not have characterized members and were not analyzed further. The remaining nine clusters were designated as subgroups and secondary nodes within these clusters were designated as families. Details on each of these nine subgroups are provided in Table 1. An expanded dendrogram that includes all of the sequence identifiers in our alignment set is provided in Figure S1. The first group (Group1) includes five subgroups named as described in Table 1: monooxygenases (MOX), 2,4-dienoyl CoA reductase (DCR), adrenodoxin reductase (ADR), glutamate synthase (GMS), and alkylhydroperoxide reductase (AHR). The second group (Group 2) includes four subgroups also named as described in Table 1: disulfide reductase (DSR), NADH peroxidase/oxidase and CoA-disulfide reductase (POR), NADH ferredoxin reductase (NFR), and NADH dehydrogenase (NDH).

**Table 1:**
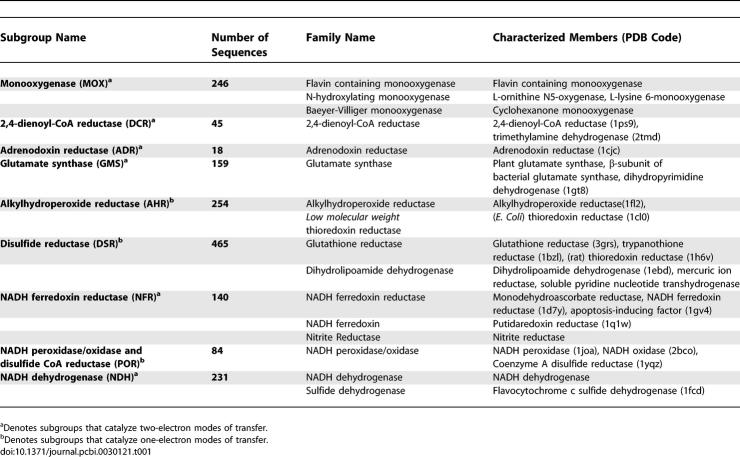
Subgroups of the tDBDF Superfamily

**Figure 4:**
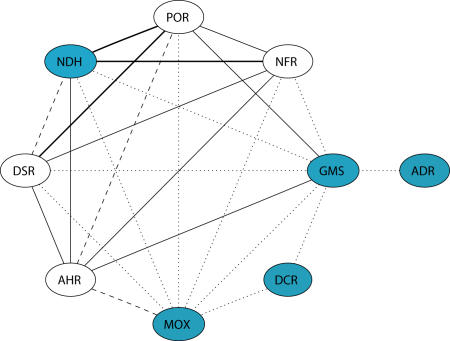
The Connectivity between Subgroups Based on E-values from Profile-Based HMM Searches Circles represent subgroups and edges represent connections between two subgroups. The strength of each connection is represented by different types of lines: dotted, E-value > 10^−10^; dashed, <10^−10^; solid, <10^−20^ and bold, <10^−30^. Blue circles designate new subgroup connections identified in this study using sequence information.

Two additional types of evidence, HMM-based connections and similarity in quaternary structure, support the relationships shown in the dendrogram (Figure 3). First, the large-scale sequence search based on HMM profiles reveals two main groups and connections between their constituent subgroups, consistent with those in the dendrogram (Figure 4). Subgroups within each group are most strongly connected to other subgroups in the same group. For example, in Group 1 the ADR subgroup is connected only to the GMS subgroup, while DCR is connected only to the MOX and GMS subgroups. Although MOX and GMS are connected to both groups through multiple subgroups, including AHR from Group 1 and NFR, DSR, and NDH from Group 2, their connections are strongest with the AHR subgroup. Subgroups within Group 2 also show the strongest connections with other subgroups in that group. For example, although the NFR subgroup is connected with both Group 1 subgroups GMS, MOX, and AHR, and Group 2 subgroups, DSR, NDH, and POR, the HMM E-values for those Group 2 subgroup connections are 10^−30^, 10^−17^, and 10^−28^ respectively, compared with E-values for connections to the Group1 subgroups of 10^−13^ (AHR), 10^−8^ (GMS), and 10^−6^ (MOX). The AHR (Group 1) and DSR (Group 2) subgroups are also multiply connected to subgroups from both groups, but again, their most statistically significant connections are to subgroups within their own group.

With respect to structural differences between the groups, each group can also be distinguished with respect to the number and organization of domain structures. All of the subgroups within Group 2 contain fused C-terminal domains that are distantly related. This domain exhibits a common fold referred to in the Protein Data Bank (PDB) as the “Carbon monoxide (CO) dehydrogenase flavoprotein C-domain-like” fold. This suggests that subgroups within Group 2 may have evolved from a common ancestor with a similar domain organization (see below for additional discussion).

### Evolutionary Relationships among tDBDFs

Our analysis of the overall sequence and structural similarities among all of the subgroups identified here as belonging to the tDBDF superfamily suggests a common evolutionary relationship that is supported by several lines of evidence. First, all contain highly conserved motifs (Figure 2) despite low pairwise sequence identity between many of the members. (Pairwise percent sequence identity is generally less than 15% between sequences in different subgroups.) Second, the dinucleotide binding domains of all known tDBDF structures define a unique fold type, classified as the FAD/NAD(P)-binding domain fold in the Structural Classification of Proteins (SCOP) database [20,21]. Third, subgroups previously identified as structural homologues (AHR, POR, NDH, ADR, and DSR) [18] are shown in Figure 4 to be connected to other subgroups (MOX, GMS, DCR, and NFR), supporting the assignment of a common evolutionary relationship. Moreover, the subgroups are multiply connected (searches with one subgroup's profile finds more than one protein from another subgroup), further indicating that they all evolved from a common ancestor.

We note that some of the tDBDF superfamily members, specifically the DSR, POR, and ADR subgroups and the cytochrome c sulfide dehydrogenase family of the NFR subgroup, have been previously classified as homologues of the GR2 group of the glutathione reductase superfamily, a set of “one dinucleotide binding domain” flavoproteins [18]. However, our sequence and structural comparisons do not find convincing links to GR2 proteins. Neither the expectation values from sequence comparisons using HMM profiles, nor Z-scores from structural comparisons using the Combinatorial Extension (CE) program [22], are statistically significant. Furthermore, the sequence similarities between the GR1 and GR2 subgroups (Dym and Eisenberg nomenclature) are limited to the FAD binding motif, a short segment in the N-terminus that comprises a stretch of fewer than thirty amino acids. The differences between our conclusions and those of Dym and Eisenberg may also reflect the possibility that these two groups may have diverged beyond recognition using homology-searching methodologies or they are related indirectly through domain swapping of the FAD-binding motif or by convergent evolution.

### The tDBDF Structural Scaffold, Cofactor Configuration, and Hydride Transfer

**Figure 5:**
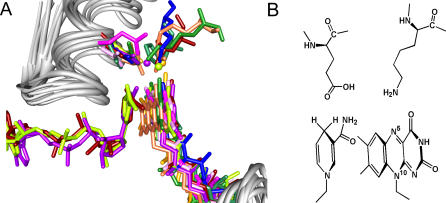
The Active Sites Structure of Members of the tDBDF Superfamily (A) The superimposed active sites of some divergent members of the tDBDF superfamily. Protein backbones are shown in gray. The cofactors and conserved sidechains important for stabilizing the isoalloxazine and nicotinamide ring complex are shown in color. The stabilizing water residues are shown as balls. Colors, PDB identifiers, and numbers of the displayed residues are as follows. DSR subgroup: dark red, glutathione reductase (1get, Glu^181^, Lys^50^); plum, mouse thioredoxin reductase (1zkq, Lys^94^, Glu^232^); and sea green, 2-ketopropyl coenzyme M oxidoreductase/carboxylase, (1mo9, Glu^228^, His^90^). NDH subgroup: magenta, flavocytochrome c sulfide dehydrogenase (1fcd, Glu^167^, N/A). NFR subgroup: blue, putidaredoxin reductase (1q1w, Glu^163^, Lys^50^). POR subgroup: chartreuse, NADH peroxidase (2npx, Glu^163^, H_2_O^490^). Group2 proteins. DCR subgroup: forest green, 2,4-dienoylCoA reductase (1ps9, Asp^508^, Lys^421^). ADR subgroup: goldenrod, adrenodoxin reductase (1e6e, Asp^159^, H_2_O^16^). AHR subgroup: purple, *E. Coli* thioredoxin reductase (1f6m, Glu^167^, H257). GMS subgroup: coral, dihydropyrimidine dehydrogenase (1gte, Asp346, Arg^235^). (Note that the structural information on the MOX subgroup was not available when this analysis was done, thus it was not represented in this figure. However, we have confirmed that it follows the same superfamily theme). (B) Illustration showing an example of common elements of the active site.

Our analyses suggest that the structural scaffold represented by all of the highly divergent members of the tDBDF superfamily evolved specifically to bind the pyridine nucleotide and FAD cofactors in a precise conformation to facilitate hydride transfer. First, each conserved motif interacts with one of these cofactors. Distributed among both dinucleotide binding domains, these motifs provide binding interactions with the FAD and pyridine nucleotide cofactors. Second, Vallon has associated the loss of dinucleotide binding with the loss of the tDBDF specific motifs, showing the importance of these motifs in binding cofactors [17]. Third, the rarity of inserts and deletions within the tDBDF scaffold and their occurrence only on the surfaces of their respective structures, far from the active sites, suggests that high precision in the geometry of binding of both cofactors is required for productive complex formation. As noted by many others (see [18] and references therein), both cofactors, anchored by conserved motifs, adopt an elongated conformation with their reactive moieties. The isoalloxazine ring of FAD and the nicotinamide ring of pyridine nucleotide are within 3.5 Å of each other, forming a stacked or endo configuration [23]. This conformation is conserved among all the tDBDFs, further supporting the experimental evidence indicating similarity in the reductive half-reaction, especially when viewed in the context of the broad variations they exhibit in both substrates and interaction partners (Figure 5). A number of studies provide evidence that this conserved orientation is optimal for hydride transfer from the pro-S position [24, 25]. Molecular orbital calculations on model compounds show that an endo configuration between the hydride donor and hydride acceptor minimizes the transition state energy [25]. This configuration results in a bent structure with an angle of 150–160 degrees between hydride acceptor, hydride, and hydride donor [24]; similar angles have been observed in other hydride transfer complexes [26,27]. The bent structure in the endo configuration maximizes the overlap between the highest occupied molecular orbital (HOMO) of the hydride ion and the owest unoccupied molecular orbital (LUMO) of the hydride acceptor, minimizing the activation energy of the hydride transfer reaction [24,25]. Similarly, the pyridine nucleotide adopts a trans conformation with respect to the carboxamide group, thought to further increase reactivity for hydride transfer [25,28,29].

Also shown in Figure 5, conserved residues within the active site of each protein provide further stabilizing interactions to the isoalloxazine ring and nicotinamide ring complex. A glutamate/aspartate residue from the pyridine nucleotide binding DBM, nearly always conserved among all the tDBDFs, is within hydrogen bonding distance of the nicotinamide ring of the pyridine nucleotide (Figures 2 and 5). The hydrogen bonding interaction between the carboxamide of the nicotinamide ring and the conserved glutamate/aspartate residue has been shown to stabilize the isoalloxazine ring and nicotinamide ring complex [30]. Second, a lysine residue with its ɛ-amino group within hydrogen bonding distance of N5 of the isoalloxazine ring of FAD is conserved among a number of subgroups (first starred residue in Figure 2). Structures from subgroups that are lacking the lysine residue have a water molecule overlapping the ɛ-amino group of the lysine, as shown in Figure 5. This lysine or water molecule may play a role in stabilizing the reduced flavin intermediate by hydrogen bonding [31]. In support of this notion, a similar mechanism involving an active-site water molecule has been proposed in adrenodoxin reductase [32]. Using a model complex, it has also been argued that the hydrogen-bonding interaction with N5 of the isoalloxazine ring is advantageous because it increases the reactivity of C4 in the isoalloxazine ring with thiols and peroxides [33,34]. The reaction mechanisms of a number of tDBDF proteins have been shown to involve C4 (see [19] and references therein).

### Evolution of Structural and Functional Variation in the tDBDF Superfamily

**Figure 6:**
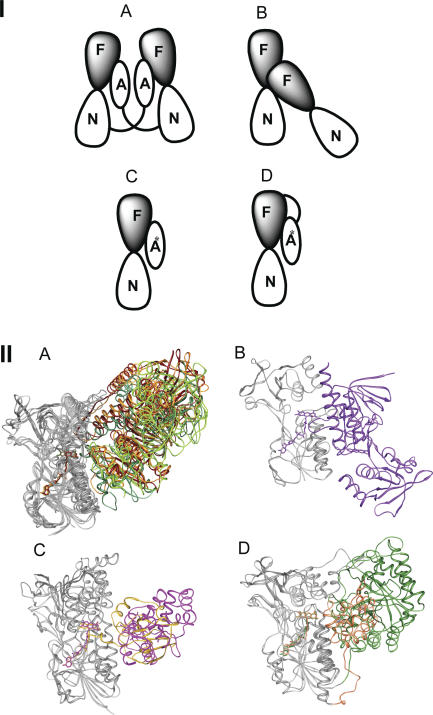
Quaternary Structures of Members of the tDBDF Superfamily (I) Illustration showing the four main types of quaternary structures observed. F, FAD; N, NAD(P)H; **A** represents a domain that assists in binding small molecules that accept two electrons and/or presents two cysteine residues that can transfer electrons to external electron acceptors; A* represents a domain or protein that accepts one electron at a time using heme or an iron-sulfur cluster. (II) Structural superimposition showing the quaternary structures of some divergent members of the tDBDF superfamily. tDBDF domains are shown in gray. The C-terminal domains of the members of the DSR and NDH subgroups are not shown for ease of viewing. Colors and PDB identifiers are as follows. A) DSR subgroup: dark red, glutathione reductase (1get); sea green, 2-ketopropyl coenzyme M oxidoreductase/carboxylase (1mo9); orange, mercuric reductase. POR subgroup: chartreuse, CoA Disulfide Reductase (1yqz). B) AHR subgroup: purple, *E Coli* thioredoxin reductase (1f6m). C) NDH subgroup: magenta, cytochrome c sulfide dehydrogenase (1fcd). ADR subgroup: goldenrod, adrenodoxin reductase (1e6e). D) DCR subgroup: forest green, 2,4-dienoylCoA reductase (1ps9). GMS subgroup: coral, dihydropyrimidine dehydrogenase (1gte).

As described in the previous section, the tDBDF scaffold is specialized to facilitate a common hydride transfer reaction. How, then, has nature generated the enormous functional diversity exhibited across the varied members of the superfamily? Our global analysis across the superfamily suggests an answer: functional variation is achieved by varying the proteins that interact with these two-dinucleotide binding domains. Thus, nature has taken advantage of the intrinsic property of FAD to accept two electrons as a hydride and transfer them either one or two electrons at a time to a wide variety of acceptor molecules and proteins to achieve diversity in the reactions that can be catalyzed. These protein–protein interactions can generally be associated with four types of quaternary structures, two types of homodimers, and two types of heterodimers (Figure 6I and II). These different types of quaternary structure are associated with different modes of electron transfer to a variety of small molecule or protein acceptors (see Figure 1). Both types of homodimeric complexes generally transfer two electrons at a time to substrates, mainly via one or multiple cysteine residues. Members of the DSR/POR and AHR subgroups form the first and second types of homodimers, respectively (Figure 6I, Figure 6II, types A and B). For example, members of the DSR subgroup, which includes glutathione reductase and the so-called “high molecular weight” thioredoxin reductase (large TR) such as that from humans, form homodimeric complexes (Figure 6I, Figure 6II, type A) in which a C-terminal extension from the second subunit interacts with the two dinucleotide binding domains of the first subunit to assist in binding and/or reducing oxidized substrates such as glutathione disulfide and thioredoxin. The second type of homodimer (Figure 6I, type B), formed by both alkylhydroperoxide reductase and the so-called “low molecular weight” thioredoxin reductase (small TR) such as that found in *Escherichia Coli (E. coli)*, is a special case and will be discussed in further detail below.

Members of the subgroups that form heterodimeric complexes generally transfer one electron directly from FAD to a small molecule one-electron acceptor, namely heme or an iron-sulfur cluster bound to an interacting protein. Members of subgroups NDH, NFR, and ADR form one type of heterodimer (Figure 6I, Figure 6II, type C) while members of the DCR and GMS subgroups form another (Figure 6I, Figure 6II, type D). The primary structural difference between heterodimeric types is that in type C, the interacting proteins are independent structures, whereas in type D, the interacting proteins, a TIM barrel in DCR and ferredoxin-like domains in the GMS subgroup, are fused to the N-terminus of the tDBDF domain. The quaternary structure of the MOX subgroup has not been elucidated, so it is not represented in this analysis.

### Constraints Imposed by Cofactor Configuration in Protein–Protein Interactions

While conscription of varied interaction partners to serve as electron acceptors illustrates the evolutionary route taken in the tDBDF superfamily to generate divergence in their overall reactions, the mode of their interactions with respect to electron transfer from the two dinucleotide binding domains is conserved, illustrating yet another feature of the interplay between conservation of active site geometry associated with the route of electron transfer from FAD and broad variation in function via interaction with different types of electron acceptors. The cofactor/acceptor complex specifies only one access point to the FAD electron transfer site, thereby imposing a unidirectional electron flow from the re*-*side to si-side of the isoalloxazine ring (we note that AHR subgroup is an exception and it is discuss in detail below). This is due to the stacked configuration of the cofactors, which, in turn, restricts the nicotinamide ring of the pyridine nucleotide to interact with the isoalloxazine ring from the re-side of FAD. This directionality of electron flow forces all of the electron acceptors to access the FAD cofactor from the si-side of the isoalloxazine ring. As a result, all protein–protein interactions between tDBDFs and acceptor proteins, including both heterodimeric and homodimeric complexes, are mediated through the face of the tDBDF that provides access to the active site from the si-side of FAD (Figure 6II). This requirement is stringent, despite wide differences in the nature and organization of protein–protein interactions across the entire tDBDF superfamily (Figure 1, Figure 6I). Thus, when tDBDFs are superimposed, all of the interacting partners (which represent a number of different fold classes), both one-electron acceptor proteins and the interacting domains of homodimers, are also superimposed with respect to the face presenting the interactions site at which electrons are received (Figure 6II).

**Figure 7:**
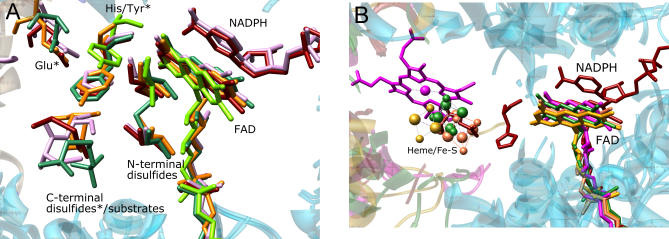
Structural Superposition Showing the Electron Transfer Route for Members of the tDBDF Superfamily (A) Two-electron transfer route. Residues designated with an asterisk * are from the C-terminal interacting domain of the second subunit of the acceptor proteins. Colors, PDB identifiers, and the numbers of the displayed residues are: dark red, 1grt, glutathione reductase (C^42^–C^47^, H^439^, E^444^, glutathione disulfide [only the disulfide bond is shown for ease of viewing]); plum, 1zkq, mouse thioredoxin reductase (C^86^–C^91^, H^143^, H^497^, E^502^, C^522^–C^523^); sea green, 1mo9, 2-ketopropyl coenzyme M oxidoreductase/carboxylase (C^82^–C^87^, F^501^, N^503^, 2-ketopropyl coenzyme M); orange, mercuric reductase (C^207^–C^212^, Y^605^, E^610^, C^628^, C^629^); chartreuse, 1yqz, CoA disulfide reductase (C^43^, Y^419^). (B) One-electron transfer route. One-electron acceptors are forest green, 2,4-dienoylCoA reductase: 4Fe–4S cluster; goldenrod, adrenodoxin reductase: 2Fe–2S cluster; coral, dihydropyrimidine dehydrogenase: 4Fe–4S cluster; magenta, cytochrome c sulfide dehydrogenase: heme. For comparison, H^439^ and E^444^ of glutathione reductase from the two-electrons transfer group also are shown.

This conservation of the mode of interaction between tDBDFs and interacting proteins is evident at an atomic level of detail. Interacting proteins or domains provide functionally important residues that assist in directing electrons from FAD to acceptors. When the tDBDFs are superimposed, the positions of the side chains of these functionally important residues and of the small molecule electron acceptors bound to interacting proteins or domains are superimposed, suggesting stringent structural constraints on these electron transfer paths. The reaction mechanism of members of the subgroup that catalyze two electrons at a time begins with the transfer of two electrons originated as hydride from pyridine nucleotide to disulfide or cysteine sulfenic acid within the tDBDF scaffold (designated as N-terminal disulfides in Figure 7A) via reduced FADH_2_. In the next step, residues from the interacting domain facilitate the transfer of these two electrons to cognate acceptors. For example, in the glutathione reductase and 2-ketopropyl coenzyme M oxidoreductase from the DSR subgroup, the interacting domain assists in anchoring the small molecule acceptors glutathione disulfide and 2-ketopropyl coenzyme M, respectively, facilitating their reduction. The disulfide bonds that accept the electrons in these substrates superimpose (designated as C-terminal disulfide/substrates in Figure 7A). Similarly, in mercuric reductase, the C-terminal interacting domain provides two cysteine residues important in binding mercuric substrates [35–37], and these superimpose with the disulfides of these small molecule substrates (designated as C-terminal disulfide/substrates in Figure 7A). Analogous to mercuric reductase, the large TR from the DSR subgroup uses two cysteine residues or cysteine and a selenocysteine residue from the C-terminal domain to shuttle electrons to thioredoxin. Although the coordinates of these two cysteine residues are not resolved in currently available crystal structures due to their high mobility, superimposition of a structure in which these two residues have been modeled [38] shows that they occupy positions that are similar to those of the cysteines in mercuric reductase and the disulfides in the small molecule acceptors (designated as C-terminal disulfide/substrates in Figure 7A).

In the DSR subgroup, two additional residues, generally a histidine and a glutamate from the C-terminal interacting domain, play an important role in catalysis. Sequence alignment shows that these two residues, which have been shown to assist in acid-base catalysis [19], are strictly conserved among the glutathione reductase, dihydrolipoamide dehydrogenase, and large TR families. In mercuric reductase and pyridine nucleotide transhydrogenase, also in the DSR subgroup, this histidine residue is replaced by a tyrosine. When those tDBDFs are superimposed, these residues from the acceptors are also superimposed (Figure 7A). Similarly, adding the POR subgroup protein CoA disulfide reductase to the superposition shows an analogous tyrosine residue superimposed with these histidine and tyrosine residues (Figure 7A). Taken together, these results provide structural evidence suggesting that the two-electron transfer mechanisms in these diverse enzymes are fundamentally similar.

**Figure 8:**
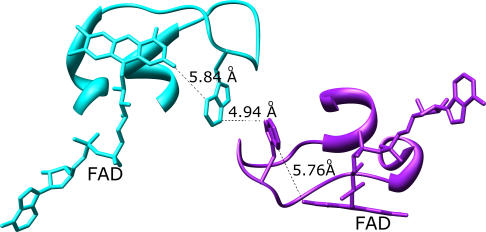
The Interaction between Subunits of Homodimeric Thioredoxin Reductase, a Member of the AHR Subgroup (*E. Coli* Thioredoxin Reductase, 1f6m) The first subunit is shown in cyan and the second is in purple. FAD and tryptophan sidechains are displayed. Distances between atoms are indicated.

Although the presence of higher-order oligomeric structure for most tDBDFs that perform two-electron transfer can be explained by the functional role contributed by these interacting partners, the role of dimer formation in the AHR subgroup is not immediately evident. As a result, these proteins have been previously considered an exception and an outlier in the superfamily [39] (Figure 6I; Figure 6II, type B). Although the AHR subgroup members also form a dimer at the same face of the tDBDF used by all of the other subgroups, unlike other subgroups the interacting domain does not play a role either as an electron acceptor or in providing assistance in substrate binding. Yet, it has been shown that these proteins are only functional as dimers [40]. Our detailed analysis of one of these subgroup members, the small TR structure (PDB 1f6m), shows that the interaction of the first FAD binding domain with the second at the tDBDF interface positions a loop from the si-side of the FAD from the first subunit to interact with the cognate loop from the second subunit. Tryptophan residues at the tips of these two loops make close contact with each other, providing a potential route for intersubunit communication (Figure 8). Interestingly, the tryptophan residue from the first subunit occupies the same relative position as that occupied by the one-electron acceptors including heme and iron-sulfur clusters (see discussion below).

When tDBDFs that transfer electrons one at a time are superimposed, all of the cognate one-electron acceptor molecules, heme or iron-sulfur clusters bound to their respective interacting proteins, are also superimposed (Figure 7B), indicative of mechanistic similarity in all the enzymes that catalyze reactions based on a one-electron transfer mechanism. Even more remarkable, the glutamate residue from the C-terminal domain of the two-electron transfer protein glutathione reductase (in the DSR subgroup discussed above) also superimposes with the one-electron acceptors (heme or iron-sulfur cluster) (Figure 7B). Thus we conclude that despite differences in whether one or two electrons are transferred at a time and wide variations in quaternary structure, homodimers and heterodimers interact with the FAD cofactor in similar ways.

### Functional Predictions

**Figure 9:**
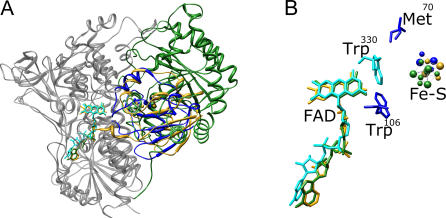
Structural Overlap of Putidaredoxin Reductase and Putidaredoxin with Analogous Redox Pairs from Different Subgroups (A) Structural superposition was performed using coordinates of putidaredoxin reductase (PDB:1q1w), adrenodoxin reductase (1e6e), and 2,4-dienoylCoA reductase (1ps9). Putidaredoxin was superimposed separately on adrenodoxin from the adrenodoxin reductase/adrenodoxin complex (1e6e). The tDBDFs are shown in cyan and the interacting proteins are shown in the following colors: putidaredoxin (PDB:1oqr), blue; adrenodoxin (PDB:1e6e), gold; and N-terminal extension of 2,4-dienoylCoA reductase (1ps9), forest green. (B) Zoomed view of the active site in A, showing one-electron acceptors and the FAD cofactor. Trp^106^ and Met^70^ from putidaredoxin are shown in blue and Trp^330^ from putidaredoxin reductase is shown in cyan.

As noted earlier, the functions of a large proportion of the proteins we have identified as members of the tDBDF superfamily have not been experimentally validated. The analysis provided here allows us to assign functions to many of these sequences as well as correct misannotated functions in public databases (S. Ojha and P. C. Babbitt, unpublished data). In addition, understanding how varied functions have evolved in the tDBDFs by diversification of protein–protein interactions allows us to predict details of some aspects of function and mechanism of less–well-characterized members of the superfamily. For instance, it has been established that members of the NADH-dependent ferredoxin reductase family from the NFR subgroup catalyze a one-electron transfer reaction, originated from NADH as a hydride ion, to one-electron acceptors including ferredoxin and putidaredoxin [41–43]. Lack of knowledge regarding the structures of the complexes formed between these redox partners has hampered elucidation of their modes of interaction and the electron transfer route from FAD to the iron-sulfur clusters. Independent superposition of putidaredoxin reductase, a member of NFR subgroup, and its electron acceptor protein putidaredoxin, on redox complexes of homologs from other subgroups (adrenodoxin reductase/adrenodoxin and 2,4-dienoyl CoA reductase/N-terminal extension of 2,4-dienoyl CoA reductase) shows that the FAD cofactors as well as the iron-sulfur clusters bound to these protein partners are also superimposed (Figure 9). This suggests that the electron transfer mechanism of members of the NFR subgroup may be similar to that of the members of the other subgroups that catalyze similar reactions (ADR and DCR). Residues Trp^330^, Val^302^ and Pro^46^ have been previously predicted to be the docking site for putidaredoxin on putidaredoxin reductase, with Trp^106^ and Met^70^ from putidaredoxin providing major hydrophobic interactions [44]. Our result compares well with these predictions and provides additional evidence that these critical residues are likely to be positioned as predicted (Figure 9B).

### Unanswered Issues in the Evolution of Diversity in the tDBDF Superfamily

Although the large-scale study presented here sheds light on how the two cofactors impose structural and functional constraints on evolutionary variations in the tDBDF superfamily, important issues about the evolution of these enzymes are yet to be elucidated. One of these, beyond the scope of this study, is the route by which the variations in oligomeric structures and protein–protein interactions evolved. Another, especially pertinent to the mechanistic issues addressed in this work, is the path by which superfamily members that transfer electrons from FAD either one or two electrons at a time evolved. The dendrogram in Figure 3 shows that members of the tDBDF superfamily that catalyze reactions based on one-electron or two-electron modes of transfer do not cluster together, suggesting that a complicated evolutionary path gave rise to these two types. An evolutionary scenario consistent with those observations suggests that transfer of electrons either one or two at a time is a promiscuous property that can be accessed by many of the tDBDF subgroups. The fact that contemporary members of the superfamily can catalyze electron transfer by either mode provides evidence for the promiscuous nature of these electron transfer modes. For example, a number of enzymes that catalyze two-electron transfer as the canonical reaction, including glutathione reductase and dihydrolipoamide dehydrogenase from the DSR subgroup and NADH peroxidase from the POR subgroup, have been shown to catalyze one-electron transfer reactions promiscuously [45–47], suggesting that these subgroups may have evolved from an ancestor capable of catalyzing reactions both one and two electrons at a time.

### Conclusion

Our analysis of more than 1,600 diverse members of the tDBDF superfamily shows that the divergence of enzyme functions has been stringently constrained by the specific organization of the cofactors in the active site. This constraint is manifested even in the details of electron transfer from FAD via protein–protein interactions, which represent the primary means by which nature has evolved variation in the overall reactions of these enzymes. The conservation in cofactor configuration that facilitates the common hydride transfer resembles the situation in mechanistically diverse enzyme superfamilies, in which a partial reaction or other chemical capability common to all divergent superfamily members specifies the underlying chemistry that the superfamily can perform. Overlaid on this common design, variation in the chemical reactions has evolved in both cases by directing a common intermediate generated in the course of the reaction to a variety of products using different interactions in the active site to support additional partial reactions that are subgroup- or family-specific. However, the mechanism for evolution of new functions in these cofactor-dependent enzymes differs from that found in cofactor-independent superfamilies in important ways. In cofactor-independent mechanistically diverse superfamilies, even the conserved structural features that facilitate the formation of common intermediates exhibit subtle variations, allowing them to use a large variety of different substrates. For example, variations in the identity of the residue used as the general base (enolase superfamily [48]), the number and configuration of divalent metal ions in the active site (amidohydrolase superfamily [4]), the position of critical catalytic residues relative to a common feature required for catalysis (crotonase superfamily [49]), and the organization of interacting domains forming the active site (haloacid dehalogenase superfamily [5,50]) have all been catalogued. In the tDBDF superfamily, by contrast, conformational variations in the geometry of the cofactors are rarely sampled because this would require altering the geometry of binding pockets required for cofactor-mediated catalysis. Although different combinations or replacement of cofactors can be envisioned (judging by the example of the tDBDF superfamily member, cytochrome c sulfide dehydrogenase, in which the pyridine nucleotide is replaced by hydrogen sulfide), this is apparently seldom sampled during evolution.

## Materials and Methods

### Identification and clustering of proteins in the tDBDF superfamily.

Sequence searches were performed using the HMMer search (http://selab.janelia.org) [51] and BLAST (http://www.ncbi.nlm.nih.gov/blast) programs [52] against the 90% identity-filtered nonredundant protein database (nrdb90, updated in July 2006) [53]. Multiple alignments and dendrograms were generated with SATCHMO [54], a sequence alignment and tree-building tool based on (HMMs). The structural alignments were generated using Chimera [55].

The sequences of structurally or experimentally characterized superfamily members previously listed by Vallon et al. [17] were obtained from the PDB (http://www.rcsb.org/pdb) or the National Center for Biotechnology Information and used as seeds in an initial sequence search to collect members of the superfamily. PDB identifiers or GI numbers of these sequences are listed under Accession Numbers in the Supporting Information at the end of this paper (and in Table 1). The structural coordinates of mercuric ion reductase and mouse thioredoxin reductase, with its C-terminal cysteine and selenocysteine modeled, were generously provided by Emil F. Pai, University of Toronto, Toronto, Ontario, Canada, and Joseph J. Barycki, University of Nebraska, Lincoln, Nebraska, respectively.

Each sequence was used in an initial BLAST search, and the resulting hit sequences were collected at an E-value cutoff of 10^−20^. These sequences were aligned using SATCHMO, and profile HMMs were built for each primary node containing an initial seed sequence. Searching the nrdb90 database using these profile HMMs identified a total of 1,749 sequences with E-values < 10^−5^. Membership in the superfamily was validated for all sequences by the presence of previously identified motifs and by inference using available structural information as described in detail in results and discussion. After 85 false positive hits were deleted, the remaining 1,664 sequences were aligned with SATCHMO. A dendrogram generated from the alignment resulted in eleven distinct clusters, and within these clusters, fifteen different nodes. A further round of HMM searches using each of these eleven clusters to generate a separate model failed to identify any additional sequences.

## Supporting Information

Figure S1 An Expanded Dendrogram That Includes All of the Sequence Identifiers in Our Alignment Set (236 KB PDF)

### Accession Numbers

The following is a list of accession numbers of seed sequences used as query, from the Protein Data Base (http://www.rcsb.org): Adrenodoxin reductase (1cjc); BaiH-Trimethylamine dehydrogenase (2tmd); Flavocytochrome c sulfide dehydrogenase, (1fcd); Glutathione reductase (1ges); NADH oxidase/NADH peroxidase (1nhr); and Rubredoxin/putidaredoxin/ferredoxin reductase, (1d7y);

The following is a list of the GI numbers of seed sequences used as query, from the GenBank NCBI database (http://www.ncbi.nlm.nih.gov/Genbank/index.html): Cyclohexanone monooxygenase (gi:27657620); Glutamate synthase (gi:146209); Lysine:N6-, ornithine:N5-hydroxylase, (gi:1073001); NADH dehydrogenase, (gi:118589); and Nitrite reductase, (gi:585562).

## Abbreviations

ADR: adrenodoxin reductase
AHR: alkylhydroperoxide reductase
DBM: dinucleotide binding motif
DCR: 2,4-dienoyl CoA reductase
DSR: disulfide reductase
FAD: flavin adenine dinucleotide
GMS: glutamate synthase
HMM: hidden Markov model
MOX: monooxygenase
NDH: NADH dehydrogenase
NFR: NADH ferredoxin reductase
PDB: Protein Data Bank
POR: NADH peroxidase/oxidase and CoA-disulfide reductase
tDBDF: two-dinucleotide–binding-domains flavoprotein
TR: thioredoxin reductase
