# Supplementary material for: Evolution of Function in the “Two Dinucleotide Binding Domains” Flavoproteins
Source: PLoS Comput Biol. 2007 Jul 20;3(7):e121. doi: 10.1371/journal.pcbi.0030121 (PMC1924876; doi:10.1371/journal.pcbi.0030121)
Supplement: Supplementary file 1 [file pcbi.0030121.sg001.pdf]

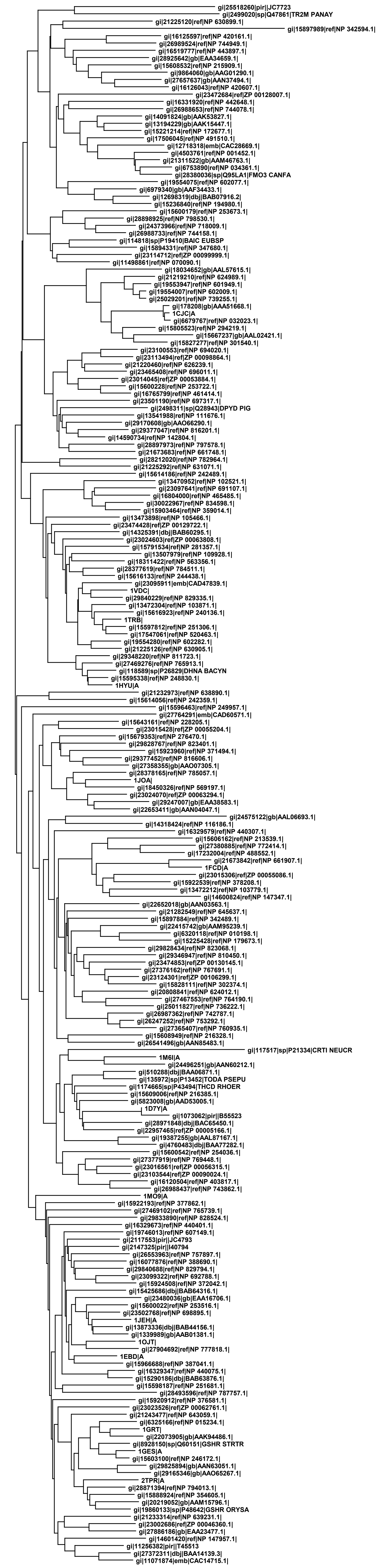

gi|25518260|pir||JC7723
gi|2499020|sp|Q47861|TR2M PANAY
gi|21225120|ref|NP 630899.1|
gi|15897989|ref|NP 342594.1|
gi|16125597|ref|NP 420161.1|
gi|26989524|ref|NP 744949.1|
gi|16519777|ref|NP 443897.1|
gi|28925642|gb|EAA34659.1|
gi|15608532|ref|NP 215909.1|
gi|9864060|gb|AAG01290.1|
gi|27657637|gb|AAN37494.1|
gi|16126043|ref|NP 420607.1|
gi|23472684|ref|ZP 00128007.1|
gi|16331920|ref|NP 442648.1|
gi|26988653|ref|NP 744078.1|
gi|14091824|gb|AAK53827.1|
gi|13194229|gb|AAK15447.1|
gi|15221214|ref|NP 172677.1|
gi|17506045|ref|NP 491510.1|
gi|12718318|emb|CAC28669.1|
gi|4503761|ref|NP 001452.1|
gi|21311522|gb|AAM46763.1|
gi|6753890|ref|NP 034361.1|
gi|28380036|sp|Q95LA1|FMO3 CANFA
gi|19554075|ref|NP 602077.1|
gi|6979340|gb|AAF34433.1|
gi|12698319|dbj|BAB07916.2|
gi|15236840|ref|NP 194980.1|
gi|15600179|ref|NP 253673.1|
gi|28898925|ref|NP 798530.1|
gi|24373966|ref|NP 718009.1|
gi|26988733|ref|NP 744158.1|
gi|114818|sp|P19410|BAIC EUBSP
gi|15894331|ref|NP 347680.1|
gi|23114712|ref|ZP 00099999.1|
gi|11498861|ref|NP 070090.1|
gi|18034652|gb|AAL57615.1|
gi|21219210|ref|NP 624989.1|
gi|19553947|ref|NP 601949.1|
gi|19554007|ref|NP 602009.1|
gi|25029201|ref|NP 739255.1|
gi|178208|gb|AAA51668.1|
1CJC|A
gi|6679767|ref|NP 032023.1|
gi|15805523|ref|NP 294219.1|
gi|15667237|gb|AAL02421.1|
gi|15827277|ref|NP 301540.1|
gi|23100553|ref|NP 694020.1|
gi|23113494|ref|ZP 00098864.1|
gi|21220460|ref|NP 626239.1|
gi|23465408|ref|NP 696011.1|
gi|23014045|ref|ZP 00053884.1|
gi|15600228|ref|NP 253722.1|
gi|16765799|ref|NP 461414.1|
gi|23501190|ref|NP 697317.1|
gi|2498311|sp|Q28943|DPYD PIG
gi|13541988|ref|NP 111676.1|
gi|29170608|gb|AAO66290.1|
gi|29377047|ref|NP 816201.1|
gi|14590734|ref|NP 142804.1|
gi|28897973|ref|NP 797578.1|
gi|21673683|ref|NP 661748.1|
gi|28212020|ref|NP 782964.1|
gi|21225292|ref|NP 631071.1|
gi|15614186|ref|NP 242489.1|
gi|13470952|ref|NP 102521.1|
gi|23097641|ref|NP 691107.1|
gi|16804000|ref|NP 465485.1|
gi|30022967|ref|NP 834598.1|
gi|15903464|ref|NP 359014.1|
gi|13473898|ref|NP 105466.1|
gi|23474428|ref|ZP 00129722.1|
gi|14325391|dbj|BAB60295.1|
gi|23024603|ref|ZP 00063808.1|
gi|15791534|ref|NP 281357.1|
gi|13507979|ref|NP 109928.1|
gi|18311422|ref|NP 563356.1|
gi|28377619|ref|NP 784511.1|
gi|15616133|ref|NP 244438.1|
gi|23095911|emb|CAD47839.1|
1VDC|
gi|29840229|ref|NP 829335.1|
gi|13472304|ref|NP 103871.1|
gi|15616923|ref|NP 240136.1|
1TRB|
gi|15597812|ref|NP 251306.1|
gi|17547061|ref|NP 520463.1|
gi|19554280|ref|NP 602282.1|
gi|21225126|ref|NP 630905.1|
gi|29348220|ref|NP 811723.1|
gi|27469276|ref|NP 765913.1|
gi|118589|sp|P26829|DHNA BACYN
gi|15595338|ref|NP 248830.1|
1HYU|A
gi|21232973|ref|NP 638890.1|
gi|15614056|ref|NP 242359.1|
gi|15596463|ref|NP 249957.1|
gi|27764291|emb|CAD60571.1|
gi|15643161|ref|NP 228205.1|
gi|23015428|ref|ZP 00055204.1|
gi|15679353|ref|NP 276470.1|
gi|29828767|ref|NP 823401.1|
gi|15923960|ref|NP 371494.1|
gi|29377452|ref|NP 816606.1|
gi|27358355|gb|AAO07305.1|
gi|28378165|ref|NP 785057.1|
1JOA|
gi|18450326|ref|NP 569197.1|
gi|23024070|ref|ZP 00063294.1|
gi|29247007|gb|EAA38583.1|
gi|22653411|gb|AAN04047.1|
gi|24575122|gb|AAL06693.1|
gi|14318424|ref|NP 116186.1|
gi|16329579|ref|NP 440307.1|
gi|15606162|ref|NP 213539.1|
gi|27380885|ref|NP 772414.1|
gi|17232004|ref|NP 488552.1|
gi|21673842|ref|NP 661907.1|
1FCD|A
gi|23015306|ref|ZP 00055086.1|
gi|15922539|ref|NP 378208.1|
gi|13472212|ref|NP 103779.1|
gi|14600824|ref|NP 147347.1|
gi|22652018|gb|AAN03563.1|
gi|21282549|ref|NP 645637.1|
gi|15897884|ref|NP 342489.1|
gi|22415742|gb|AAM95239.1|
gi|6320118|ref|NP 010198.1|
gi|15225428|ref|NP 179673.1|
gi|29828434|ref|NP 823068.1|
gi|29346947|ref|NP 810450.1|
gi|23474853|ref|ZP 00130145.1|
gi|27376162|ref|NP 767691.1|
gi|23124301|ref|ZP 00106299.1|
gi|15828111|ref|NP 302374.1|
gi|20808841|ref|NP 624012.1|
gi|27467553|ref|NP 764190.1|
gi|25011827|ref|NP 736222.1|
gi|26987362|ref|NP 742787.1|
gi|26247252|ref|NP 753292.1|
gi|27365407|ref|NP 760935.1|
gi|15608949|ref|NP 216328.1|
gi|26541496|gb|AAN85483.1|
gi|117517|sp|P21334|CRTI NEUCR
1M6I|A
gi|24496251|gb|AAN60212.1|
gi|510288|dbj|BAA06871.1|
gi|135972|sp|P13452|TODA PSEPU
gi|1174665|sp|P43494|THCD RHOER
gi|15609006|ref|NP 216385.1|
gi|5823008|gb|AAD53005.1|
1D7Y|A
gi|1073062|pir||B55523
gi|28971848|dbj|BAC65450.1|
gi|22957465|ref|ZP 00005166.1|
gi|19387255|gb|AAL87167.1|
gi|4760483|dbj|BAA77282.1|
gi|15600542|ref|NP 254036.1|
gi|27377919|ref|NP 769448.1|
gi|23016561|ref|ZP 00056315.1|
gi|23103544|ref|ZP 00090024.1|
gi|16120504|ref|NP 403817.1|
gi|26988437|ref|NP 743862.1|
1MO9|A
gi|15922193|ref|NP 377862.1|
gi|27469102|ref|NP 765739.1|
gi|29833890|ref|NP 828524.1|
gi|16329673|ref|NP 440401.1|
gi|19746013|ref|NP 607149.1|
gi|2117553|pir||JC4793
gi|2147325|pir||I40794
gi|26553963|ref|NP 757897.1|
gi|16077876|ref|NP 388690.1|
gi|29840688|ref|NP 829794.1|
gi|23099322|ref|NP 692788.1|
gi|15924508|ref|NP 372042.1|
gi|15425686|dbj|BAB64316.1|
gi|23480036|gb|EAA16706.1|
gi|15600022|ref|NP 253516.1|
gi|23502768|ref|NP 698895.1|
1JEH|A
gi|13873336|dbj|BAB44156.1|
gi|1339989|gb|AAB01381.1|
1OJT|
gi|27904692|ref|NP 777818.1|
1EBD|A
gi|15966688|ref|NP 387041.1|
gi|16329347|ref|NP 440075.1|
gi|15290186|dbj|BAB63876.1|
gi|15598187|ref|NP 251681.1|
gi|28493596|ref|NP 787757.1|
gi|15920912|ref|NP 376581.1|
gi|23023526|ref|ZP 00062761.1|
gi|21243477|ref|NP 643059.1|
gi|6325166|ref|NP 015234.1|
1GRT|
gi|22073905|gb|AAK94486.1|
gi|8928150|sp|Q60151|GSHR STRTR
1GES|A
gi|15603100|ref|NP 246172.1|
gi|29825894|gb|AAN63051.1|
gi|29165346|gb|AAO65267.1|
2TPR|A
gi|28871394|ref|NP 794013.1|
gi|15888924|ref|NP 354605.1|
gi|20219052|gb|AAM15796.1|
gi|19860133|sp|P48642|GSHR ORYSA
gi|21233314|ref|NP 639231.1|
gi|23002686|ref|ZP 00046360.1|
gi|27886186|gb|EAA23477.1|
gi|14601420|ref|NP 147957.1|
gi|11256382|pir||T45513
gi|27372311|dbj|BAA14139.3|
gi|11071874|emb|CAC14715.1|
